# Eight respectively nine out of ten patients return to sport and work after distal femoral osteotomy

**DOI:** 10.1007/s00167-018-5206-x

**Published:** 2018-10-22

**Authors:** Alexander Hoorntje, Berbke T. van Ginneken, P. Paul F. M. Kuijer, Koen L. M. Koenraadt, Rutger C. I. van Geenen, Gino M. M. J. Kerkhoffs, Ronald J. van Heerwaarden

**Affiliations:** 1 Department of Orthopaedic Surgery, Academic Medical Center, University of Amsterdam, Amsterdam Movement Sciences, Meibergdreef 9, 1105 AZ Amsterdam, The Netherlands; 2 Academic Center for Evidence-Based Sports Medicine (ACES), Amsterdam, The Netherlands; 3 Amsterdam Collaboration on Health and Safety in Sports (ACHSS), AMC/VUmc IOC Research Center, Amsterdam, The Netherlands; 4 Department of Orthopaedic Surgery, Maartenskliniek Woerden, Woerden, The Netherlands; 5 Coronel Institute of Occupational Health, Amsterdam Public Health Research Institute, Academic Medical Center, University of Amsterdam, Amsterdam, The Netherlands; 6 Department of Orthopaedic Surgery, Foundation FORCE (Foundation for Orthopaedic Research Care and Education), Amphia Hospital, Breda, The Netherlands; 7 Centre for Deformity Correction and Joint Preserving Surgery, Kliniek ViaSana, Mill, The Netherlands

**Keywords:** Distal femoral osteotomy (DFO), Supracondylar osteotomy (SCO), Lateral opening wedge, Medial closing wedge, De-rotation osteotomy, Return to sport, Return to work, Participation, Prognosis

## Abstract

**Purpose:**

Distal femoral osteotomy (DFO) is a well-accepted procedure for the treatment of femoral deformities and associated symptoms including osteoarthritis, especially in younger and physically active patients in whom knee arthroplasty is undesirable. Still, there is an apparent need for evidence on relevant patient outcomes, including return to sport (RTS) and work (RTW), to further justify the use of knee osteotomy instead of surgical alternatives. Therefore, the purpose of the present study was to investigate the extent and timing of patients’ RTS and RTW after DFO.

**Methods:**

This monocentre, retrospective cohort study included consecutive DFO patients, operated between 2012 and 2015. Out of 126 eligible patients (18–70 years, 63% female), all patients responded, and 100 patients completed the questionnaire. Median follow-up was 3.4 years (range 1.5–5.2). The predominant indication for surgery was symptomatic unicompartmental osteoarthritis and valgus or varus leg alignment caused by a femoral deformity. The primary outcome measure was the percentage of RTS and RTW. Secondary outcome measures included time to RTS/RTW, sports level and frequency, the median pre-symptomatic and postoperative Tegner activity score (1–10, higher is more active) and the postoperative Lysholm score (0–100, higher is better).

**Results:**

Out of 84 patients participating in sports preoperatively, 65 patients (77%) returned to sport postoperatively. Forty-six patients (71%) returned to sports within 6 months. Postoperative participation in high-impact sports was possible though less frequent compared to preoperative participation. Out of 80 patients working preoperatively, 73 (91%) returned to work postoperatively, of whom 59 patients (77%) returned within 6 months. The median pre-symptomatic Tegner activity score [4.0 (range 0–10)] was significantly higher (*p* < 0.01) than the reported Tegner score at follow-up [3.0 (range 0–10)]. The mean Lysholm score at follow-up was 68 (± 22). No significant differences were found between the osteoarthritis- and non-osteoarthritis group.

**Conclusion:**

Eight out of ten patients return to sport and nine out of ten patients return to work after DFO. These are clinically relevant findings, because they further justify DFO as a surgical alternative to KA in young, active knee OA patients who wish to return to high activity levels.

**Level of evidence:**

Retrospective cohort study, Level III.

**Electronic supplementary material:**

The online version of this article (10.1007/s00167-018-5206-x) contains supplementary material, which is available to authorized users.

## Introduction

Knee osteoarthritis (OA) is increasingly observed in active patients who are still of working age [20]. This population represents a challenge, because knee replacement is undesirable, given the three- to fivefold increased risk of revision surgery in young and active patients, compared to patients above the age of 55–65 [26]. Furthermore, meeting younger patients’ expectations is difficult, because their expectations tend to be higher than what a knee arthroplasty (KA) can deliver [1, 24]. Therefore, knee osteotomy has regained interest from surgeons who are looking for joint preserving alternatives to KA, resulting in a considerable increase in knee osteotomy surgery in the last decade [11, 28]. In cases of early-stage unicompartmental knee OA with a femoral deformity, distal femoral osteotomy (DFO) is considered the preferred treatment [10]. DFO is also a well-accepted procedure for the treatment of symptomatic unicompartmental overload and congenital malformations, especially in younger and physically active patients [6, 10, 13, 14, 35].

Yet, there is an apparent need for robust evidence on relevant patient outcomes, including return to sport (RTS) and return to work (RTW), to further justify the use of knee osteotomy instead of surgical alternatives [6, 33]. Systematic reviews on RTS and RTW after knee osteotomy showed that up to 85% of patients can RTS and RTW after high tibial osteotomy (HTO) [5, 16]. However, data on RTS and RTW after DFO are sparse. One study on varising DFO for lateral compartment OA, found that 23 of 26 patients returned to work, and 14 of 15 patients returned to their preoperative sports activities [4]. Another study, including 13 young athletes treated with varising DFO for symptomatic lateral compartment overload, found that all patients returned to sport at 2 years follow-up [31]. However, both studies described a small number of patients selected based on strict inclusion criteria, thus limiting generalizability. Furthermore, no studies on RTS and RTW have been performed in patients with DFOs other than varus-producing osteotomies. Finally, timing of return to sport and work after DFO has not been described previously. Both the extent and timing of RTS and RTW represent valuable information to the patient and the orthopaedic surgeon, that could be used to guide preoperative patient counselling, shared decision making and expectation management [2].

Therefore, the purpose of the present study was to investigate the extent and timing of patients’ return to sport and work after DFO in a large cohort with different indications for distal femoral corrections. This is clinically relevant information, that may be used when counselling young, active patients to discuss their expectations regarding postoperative sport and work ability after DFO. If a return to sports and work is indeed possible after DFO, this would further justify the use of DFOs in this population. Our hypothesis was that most patients return to sport and work, including high-impact activities, after DFO.

## Materials and methods

A monocentre cross-sectional study was performed in consecutive DFO patients operated on between 2012 and 2015. Eligible patients were between 18 and 70 years of age at follow-up. Patients had to understand the Dutch language and were required to be mentally able to complete the questionnaire. Patients who were treated with DFO bilaterally were asked to complete the questionnaire for the most recent operation. Eligible patients received a questionnaire by postal mail, followed by a maximum of two telephone reminders.

### Patient characteristics

Patients’ age, BMI (kg/m^2^) and education level were asked. In addition, patients were asked if they had experienced postoperative complications and whether they had been operated on the same leg again following DFO (e.g., revision surgery or knee arthroplasty). The ASA classification, degree of correction and additional information on possible revision surgery and hardware removal were collected from the electronic medical record.

### Participants

**Fig. 1:**
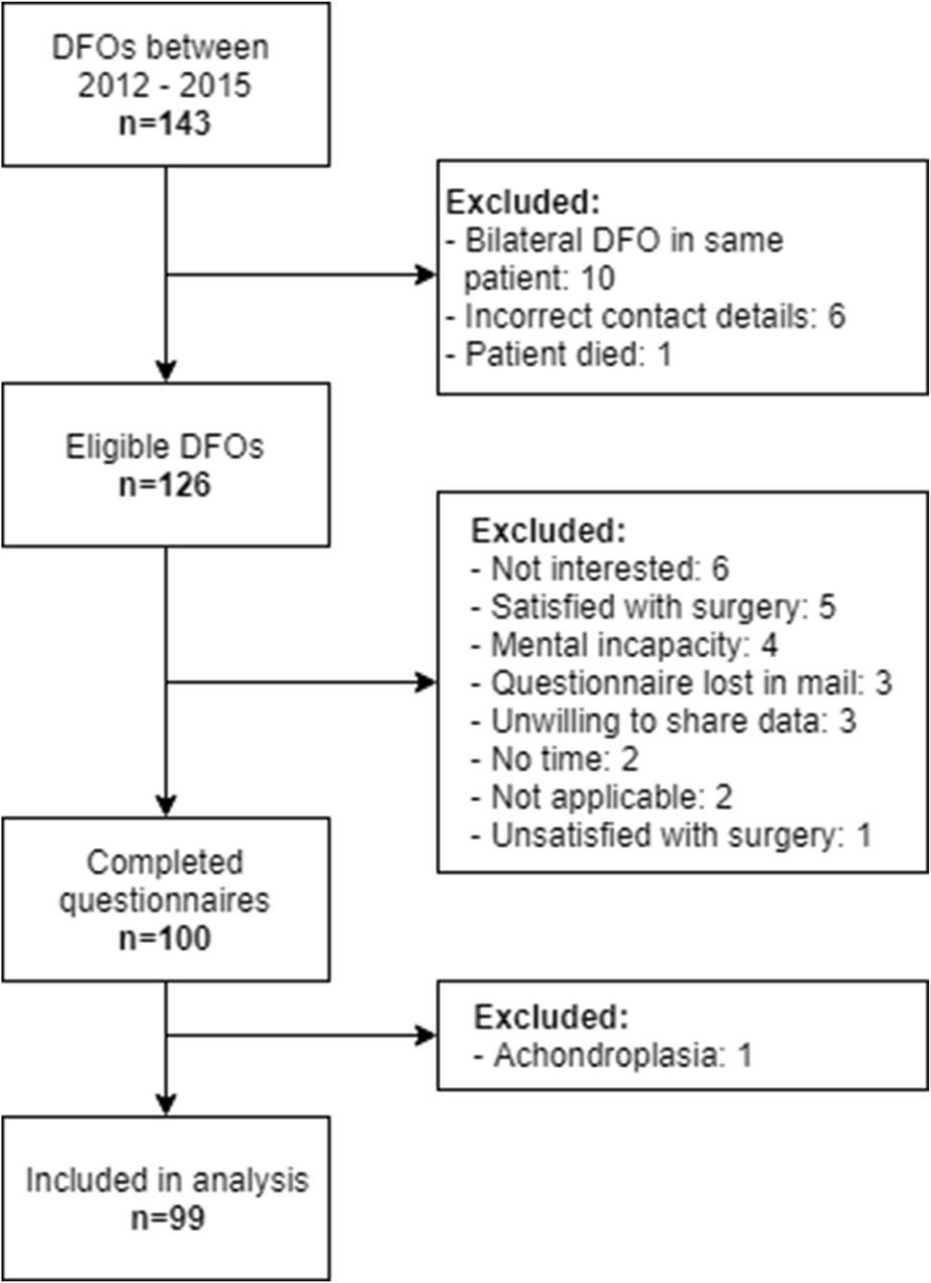
Inclusion flowchart

Out of 143 consecutive DFOs, 126 were eligible for inclusion and these patients were sent a questionnaire. All patients responded and 100 patients completed the questionnaire at a median follow-up of 3.5 years (range 1.4–5.2). One additional patient was excluded after completing the questionnaire, because she suffered from achondroplasia and had never worked or performed sports in her life. Figure 1 presents the in- and exclusion flow chart for this study. The predominant indication for surgery was symptomatic unicompartmental osteoarthritis. In addition, patients with a valgus or varus leg alignment caused by a femoral deformity without the presence of OA and patients with symptomatic rotational deformities of the femur were included. Finally, patients with a flexion contracture were treated with an extending DFO. Out of a total of 99 patients, 29 patients with a multiplane deformity or a concomitant tibial deformity were treated with combined osteotomies of the femur and tibia.

### Surgical technique and rehabilitation

**Fig. 2:**
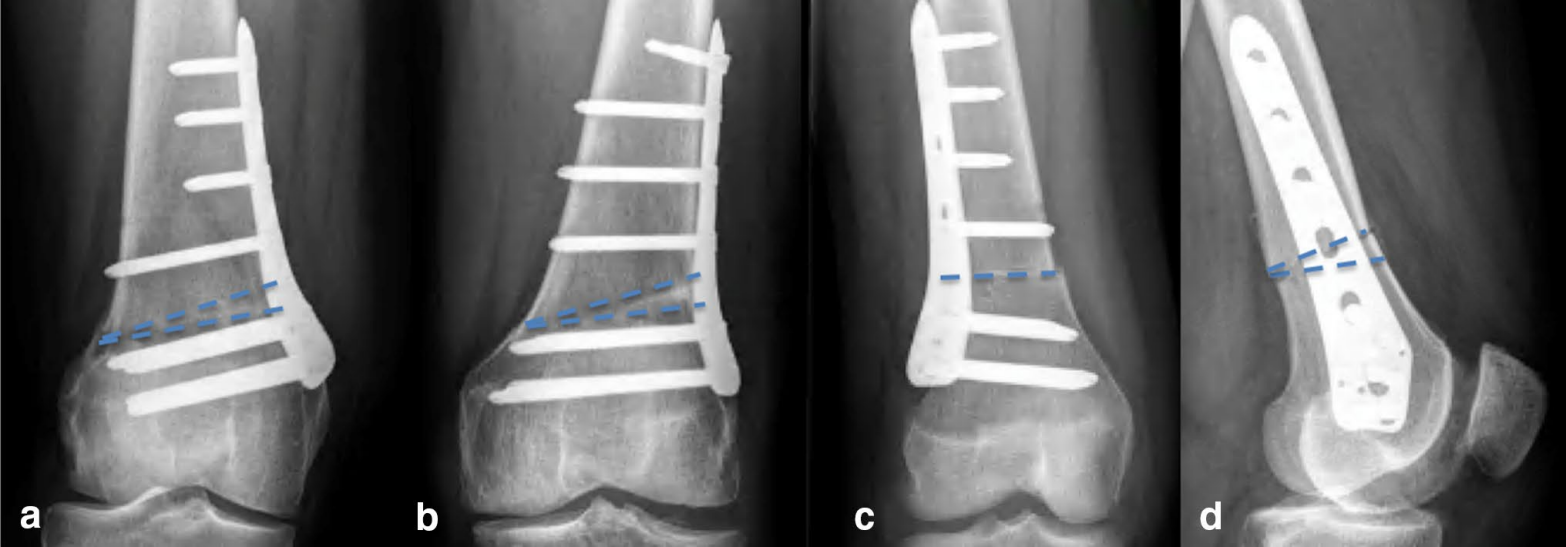
Posteoperative anteroposterior/lateral radiographs of distal femoral osteotomies (DFOs) with projected osteotomy cuts (striped lines). **a** Right knee after medial closing wedge DFO, **b** Left knee after lateral closing wedge DFO, **c** Right knee after de-rotation DFO, **d** Left knee after anterior closing wedge DFO

Surgery was performed by one of three dedicated knee osteotomy surgeons with 5–15 years of experience with DFO. The DFO frontal plane and transverse plane techniques have been described in previous publications [13, 14], and all techniques including the sagittal plane technique are illustrated in Fig. 2. For valgus malalignment, patients underwent a biplanar medial closing wedge osteotomy or a biplanar lateral opening wedge osteotomy. For varus malalignment, patients underwent a biplanar lateral closing wedge osteotomy. In case of additional valgus or varus malalignment of the tibia, a combined DFO and HTO were performed. Patients with rotational malalignment of the femur were treated with a single plane, de-rotation transverse DFO. Patients with an additional rotational malalignment of the tibia were also treated with a de-rotation transverse proximal tibial osteotomy. Finally, in case of a flexion contracture, patients were treated with a single plane extension DFO. Prior to surgery, a detailed planning was performed for each patient. Degrees of correction in frontal and sagittal plane were converted to millimetres of wedge to be resected, as measured on the calibrated radiographs. In the OR, callipers and rulers were used to define the wedge in the bone with K-wires under fluoroscopic guidance. Transverse plane corrections were calculated from standardized CT-scans. Intra-operatively, a tracker specifically designed for rotational measurements is used, together with K-wires defining the angle of rotation in the bone or to measure the angle of correction. Plate fixation in all patients was performed with angle stable plates (TomoFix, Synthes GmbH, Solothurn, Switzerland). Postoperatively, physiotherapy guided immediate range of motion exercises and muscle strengthening was started and all patients were restricted to partial weight bearing for 6 weeks. Thromboembolic prophylaxis, i.e., Enoxaparin 40 mg, was prescribed once daily for 6 weeks. After 6 weeks, knee radiographs were obtained to verify the degree of correction and to check for hardware complications. Progressive weight bearing was allowed thereafter, up to full weight bearing at 3 months. At 3 months postoperative, knee radiographs and full-length standing radiographs were obtained to verify bone healing and the correction of deformity.

### Sport outcome measures

The primary outcome measure was the percentage of patients that returned to sport postoperatively. Secondary outcome measures included the timing of RTS, the frequency, duration and type of performed sport activities pre- and postoperatively. No validated questionnaire exists to assess RTS in knee osteotomy patients. Therefore, a questionnaire was developed, based on the sports questionnaire described by Naal et al. in 2007, to investigate RTS after hip resurfacing arthroplasty and unicompartmental knee arthroplasty (UKA) [22, 23]. This questionnaire has been used in several other studies investigating RTS after knee surgery, including studies in knee osteotomy patients [8, 25]. The questionnaire ascertains patients’ preoperative and postoperative engagement in 20 sports, e.g., cycling, jogging, golf and tennis. For the present study, 14 sports were added to the questionnaire (Supplementary material 1). Preoperative sports participation was defined as both pre-symptomatically, i.e., before the onset of restricting knee symptoms, and 1 year preoperatively. Postoperative sports participation was defined as 1 year postoperatively and at final follow-up. For each selected sport, patients reported at which of those four timepoints they had participated in that sport. For each timepoint, the highest level of participation (recreative, competitive or professional) was asked. Next, sports frequency (0–7 times per week), duration (hours per week) and timing of RTS (weeks) were asked. To assess the level of impact, sports activities were rated as low-, intermediate- or high-impact according to the classification by Vail et al. [29]. In addition, patients were asked to rate their sports ability at follow-up, compared to the best sports ability in their lifetime with the following five answering categories: much worse; worse; unchanged; improved; much improved. Finally, the Tegner activity score and the Lysholm score, which have been recently validated in Dutch [7], were collected. Patient was asked to report their pre-symptomatic Tegner score and their Tegner score at follow-up. The Lysholm score was only completed for the situation at follow-up [3].

### Work outcome measures

The primary work outcome measure was the percentage of patients that returned to work postoperatively. The secondary outcome measure was the timing of RTW. First, patients were asked if they worked before the onset of restricting knee symptoms, and within 3 months preoperatively. Job title was recorded and classified as light, medium or heavy by two occupational experts, who independently scored all jobs based on work-related physical demands on the knee [19, 30]. The hours per week that patients worked 3 months preoperatively, 1 year postoperatively and at follow-up were also asked. In addition, time to RTW and changes in work load (lower; unchanged; higher) were asked. If patients did not RTW, reasons for no RTW were asked. Finally, the validated WORQ questionnaire was used to assess the impact of DFO on work-related activities [9, 18]. The WORQ consists of 13 knee-burdensome activities (e.g., kneeling, lifting/carrying, climbing stairs). Patients grade the difficulty they experience when performing each activity on a five-point Likert scale, with 0 meaning no difficulty and 4 meaning extreme difficulty/unable to perform. Patients were asked to retrospectively grade the difficulty at three timepoints: 3 months preoperatively, 1 year postoperatively and at final follow-up.

Institutional Review Board approval was obtained from the local medical ethical review board (Academic Medical Center Amsterdam, reference number W17_382 #17.448) prior to initiation of this study. All patients provided written informed consent.

### Statistical analysis

Demographic data, pre- and postoperative sport participation and work status were analysed using descriptive statistics. In addition, timing of RTS and RTW, and frequency and duration of sports participation were analysed with descriptive statistics. RTS was calculated by selecting all patients that participated in one or more sports preoperatively and calculating which percentage of these patients could RTS 1 year postoperatively and/or at final FU. The unpaired *T* test was used to compare pre-symptomatic and postoperative Tegner scores. The WORQ scores at three timepoints were dichotomized to determine how many patients experienced severe difficulty with a work-related knee-demanding activity. “Severe difficulty” and “extreme difficulty/unable to perform” were classified as “severe difficulty”. “Moderate difficulty,” “mild difficulty” and “no difficulty” were classified as “no severe difficulty”. In addition to the primary analyses for the total group, subgroup analyses for RTS and RTW were performed for the OA patients and the non-OA patients using the Chi-square test. A *p* value of *p* < 0.05 was considered significant. All statistical analyses were performed with SPSS for Windows (Version 24.0. Armonk, NY: IBM Corp.).

## Results

Table 1 presents the baseline characteristics of the total group, and of the OA- and non-OA subgroups. No intra-operative complications were encountered. There were four postoperative complications that required revision surgery: one case of a broken plate, one case of a broken and protruding screw, one case of delayed union and one case of non-union. Table 2 presents the operation type and degree of correction for the included patients.

**Table 1:** Baseline characteristics of total group and of the OA- and non-OA subgroups

Outcome measure	Total group (*n* = 99)	OA group (*n* = 64)	Non-OA group (*n* = 35)
Mean age at surgery, years (SD)	41.2 (14.2)	48.5 (8.7)	28.1 (12.9)
Median follow-up, years (range)	3.4 (1.4–5.2)	3.5 (1.4–5.2)	3.4 (1.5–5.2)
Sex, female (%)	62 (63)	39 (61)	23 (66)
Mean BMI, kg/m^2^ (SD)	27.3 (4.6)	28.4 (4.1)	25.2 (4.9)
Side, right (%)	54 (55)	40 (63)	14 (40)
ASA classification, *n* (%)			
I	67 (68)	41 (64)	26 (74)
II	31 (31)	22 (35)	9 (26)
III	1 (1)	1 (2)	–
Type of femoral deformity			
Varus	25 (25)	18 (28)	7 (20)
Valgus	58 (59)	46 (72)	12 (34)
Rotational	13 (13)	–	13 (37)
Extending	3 (3)	–	3 (9)
Revision osteotomy, yes (%)	4 (4)	3 (5)	1 (3)
Hardware removal, yes (%)	65 (66)	37 (59)	28 (80)
Timing of hardware removal, years (SD)	1.0 (0.8)	1.1 (0.8)	0.9 (0.6)

*ASA* American Society of Anaesthesiologists, *BMI* body mass index, *OA* osteoarthritis

**Table 2:** Operation type and degree of correction

Operation type	Patients (*n* (%))	Degree of correction (mean ± SD)
Medial cwDFO	42 (43%)	7.9° ± 2.9°
Lateral cwDFO	14 (14%)	6.5° ± 2.2°
Lateral owDFO	5 (5%)	7.0° ± 3.6°
Lateral cwDFO + medial owHTO	9 (9%)	6.3° ± 2.8° + 6.7° ± 1.6°
Medial cwDFO + medial cwTKO	13 (13%)	7.9° ± 4.0° + 7.5° ± 2.7°
FDO^a^	6 (6%)	18.3° ± 11.8°
FDO + TDO^a^	7 (7%)	13.9° ± 3.5° + 16.5° ± 2.3°
Extending DFO	3 (3%)	8.5° ± 5.7°

*cw* closing wedge, *DFO* distal femoral osteotomy, *FDO* femoral de-rotation osteotomy, *HTO* high tibial osteotomy, *ow* opening wedge, *TDO* tibial de-rotation osteotomy

^a^Degrees of rotational correction are presented

### Return to sport

**Fig. 3:**
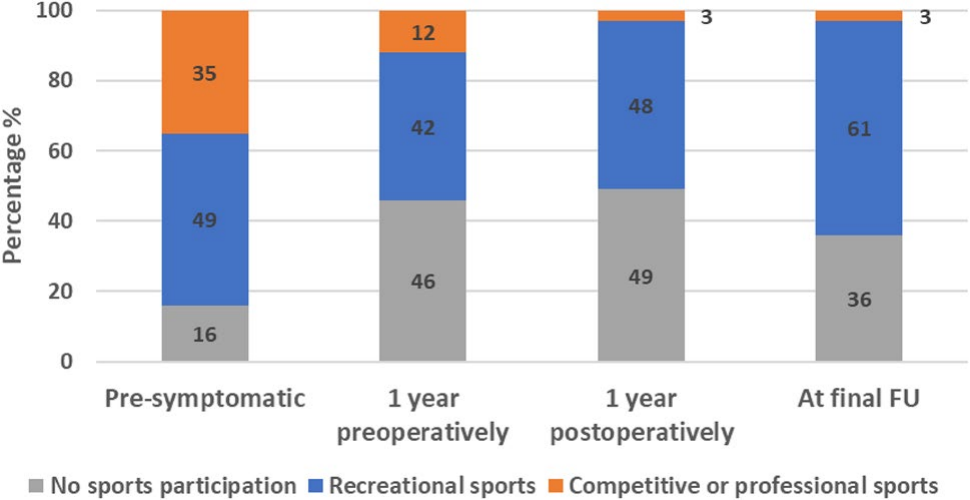
Level of sports participation (no participation, recreational or competitive/professional sports participation) of the total group at four timepoints

Out of 84 patients participating in one or more sports preoperatively, 65 patients (77%) returned to sport postoperatively. Time to RTS was ≤ 6 months in 71% of patients. In addition, four patients (4%) started participating in one or more sports postoperatively. For the OA group, 44 out of 54 patients (82%) could RTS compared to 21 out of 30 patients (70%) for the non-OA group (n.s.). Figure 3 presents the level of sports participation at four timepoints for the total group, showing a shift over time from a competitive/professional level to a recreational level. Compared to pre-symptomatically, sports frequency was lower 1 year pre- and postoperatively (Supplementary material 2). At final follow-up, frequency had increased again, but did not reach the pre-symptomatic level. A shift was found from high- to intermediate- and low-impact sports (Supplementary material 2). Sports ability at final follow-up compared to best lifetime sports ability was worse or much worse in 55 patients (60%), unchanged in 19 patients (20%) and improved or much improved in 19 patients (20%). The median Tegner score decreased from 4.0 (range 0–10) pre-symptomatically to 3.0 (range 0–10) at final follow-up (*p* < 0.01). The mean Lysholm score at final follow-up was 68 (± 22). In total, 42% of patients reported a Lysholm score of < 65 points (poor), 28% a score of 65–83 (fair), 23% a score of 84–94 (good) and 7% a score of > 94 (excellent).

### Return to work

**Fig. 4:**
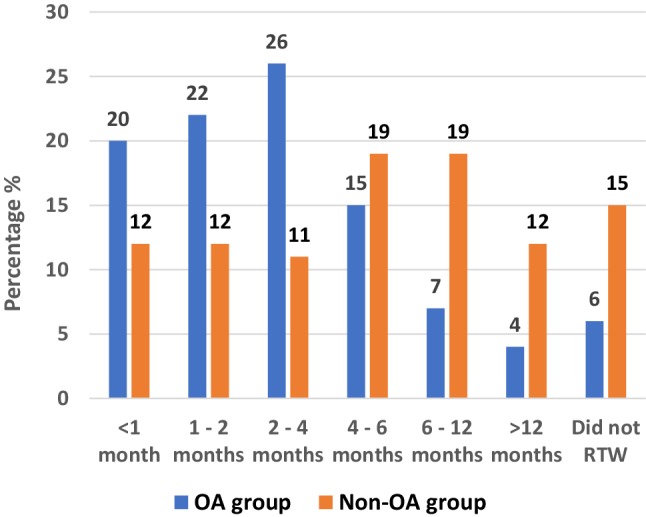
Timing of return to work for the OA group and the non-OA group

**Fig. 5:**
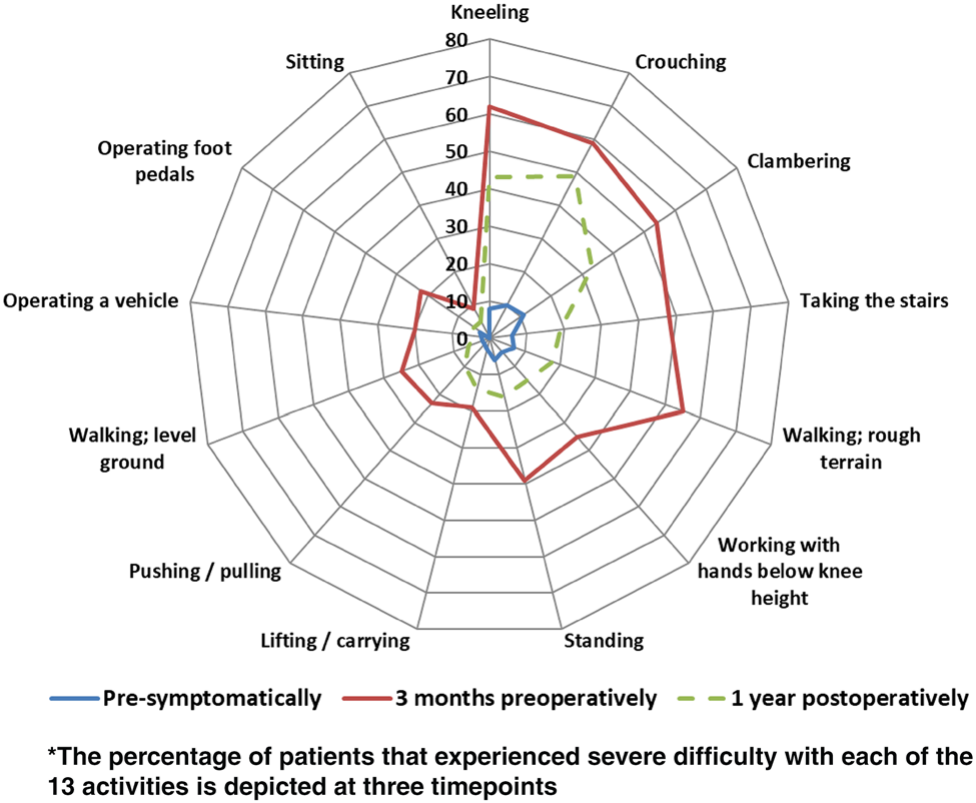
Reported difficulty with work-related tasks of the total group at three timepoints*

Before the onset of restricting knee symptoms, 80 patients (81%) were working, and 77 of them (77%) were still working 3 months preoperatively. Postoperatively, 73 out of 80 patients (91%) could RTW of whom 59 patients (81%) returned within 6 months. In addition, three patients started working postoperatively. In the OA group, 51 out of 54 patients (94%) could RTW, compared to 22 out of 26 patients (85%) in the non-OA group (n.s.) (Fig. 4). On average, patients worked an equal number of hours 1 year postoperatively compared to preoperatively and worked slightly more hours at final follow-up (Table 3). Table 4 presents the pre-symptomatic and preoperative workload, and postoperative changes in workload. Out of seven patients that did not RTW, four patients did not return due to knee complaints and three patients did not return due to physical complaints unrelated to their knee. Finally, Fig. 5 presents the WORQ scores at three timepoints. 3 months preoperatively, > 50% of patients experienced severe difficulty with kneeling, crouching, clambering and walking on rough terrain. Postoperatively, an improvement was observed for all activities. Walking on rough terrain and taking the stairs showed the largest improvement, while patients experienced most difficulty with kneeling and crouching.

**Table 3:** Number of working hours of the total group at three timepoints

	1 year preoperatively [*n* (%)]	1 year postoperatively [*n* (%)]	At final follow-up [*n* (%)]
0–8 h/wk	13 (16)	12 (16)	8 (11)
9–16 h/wk	10 (13)	12 (16)	10 (13)
17–24 h/wk	9 (11)	11 (14)	9 (12)
25–32 h/wk	12 (15)	13 (17)	15 (20)
33–40 h/wk	21 (27)	16 (21)	20 (27)
> 40 h/wk	14 (18)	12 (16)	13 (17)

*h* hours, *wk* week

**Table 4:** Preoperative knee-demanding workload and postoperative changes in workload

Workload	Pre-symptomatically (%)	Preoperatively (%)	Change in workload	1 year postoperatively (%)
Light	66	73	Lighter	14
Intermediate	25	24	Equal	79
High	9	3	Higher	7

## Discussion

The most important findings of the present study were that 77% of patients could RTS after DFO, of whom 71% returned within 6 months. Furthermore, 91% of patients could RTW, of whom 81% returned within 6 months. There was no statistically significant difference in RTS and RTW between the subgroups of OA patients and non-OA patients.

The present study is the first to assess sports participation pre-symptomatically, 1 year pre- and postoperatively and at final follow-up, allowing for a good comparison of the effect of DFO on sports ability over time. Based on previous research [16, 32], a return to the pre-symptomatic sports level was considered unlikely for most patients. This was confirmed by the reported sports ability at final follow-up, which was worse or much worse in 60% of patients compared to their best lifetime sports ability. Still, 45% performed sports ≥ 2 times/week and 41% performed sports for ≥ 3 h/week. Finally, 45% of all sports activities performed at follow-up were intermediate- or high-impact activities. Compared to HTO, DFO patients showed a lower participation in high-impact activities (10 vs. 6%) and higher participation in intermediate-impact activities (32 vs. 39%) [16]. Nevertheless, participation in intermediate- and high-impact sports was considerably higher than after TKA (11%) and UKA (23%) [34]. This might be explained by more liberal surgeons’ advice as well as higher functional benefits after DFO compared to KA, given the fact that native knee structures are preserved [6].

In addition, the present study is the first to report time to RTS after DFO. Half of the patients returned within 15 weeks and 71% returned within 6 months. Thus, 29% of patients took longer than 6 months to RTS. In TKA, average time to RTS was 13 weeks, compared to 12 weeks in UKA [34]. Therefore, DFO appears to show a functional benefit from retaining native knee kinematics, allowing demanding functional loading that would otherwise jeopardize the survival of a KA [6, 35]. In contrast, time to RTS might be somewhat longer after DFO due to the extended rehabilitation following knee osteotomy [10].

Regarding RTW, almost all patients (91%) returned to work, which is high compared to reported numbers for surgical alternatives. Average RTW in HTO patients is 85% [16], and varies between 70 and 89% in TKA patients [15, 17, 21]. Yet, it must be noted that the mean age in our cohort was comparable to studies in HTO patients, and lower compared to studies in TKA patients. Furthermore, our study is the first to report time to RTW after DFO and found that 71% returned within 6 months. This is in line with findings in HTO patients, where the mean time to RTW was 16 weeks [16]. Given the higher mean age of the OA subgroup (49 vs. 28 years) and the presence of debilitating knee OA, it is remarkable that more patients appeared to return to work and time to RTW appeared shorter compared to the non-OA group. A possible explanation is that bone healing and functional recovery are faster after DFO for unicompartmental OA, compared to de-rotation osteotomies for rotational malalignment and combined femoral and tibial osteotomies, which were mainly performed in the non-OA group [10, 12, 13]. Concerning knee-demanding work activities, as anticipated, preoperatively patients experienced most difficulty with kneeling, crouching, clambering, walking on rough terrain and taking the stairs. One year postoperatively, the number of patients experiencing severe difficulties had decreased markedly for all work-related activities, except for crouching. These findings are consistent with those in TKA patients, who experienced severe difficulty with kneeling, crouching, clambering and taking the stairs preoperatively [17]. However, at 2–3 years follow-up, the total percentage of KA patients experiencing difficulties was higher for all activities, except for crouching, compared to DFO [17, 27]. These findings indicate that DFO may provide equal or better work-related functional outcomes compared to KA.

Given the limited number of studies on RTS and RTW after DFO, a comparison with previous literature is difficult. De Carvalho et al. found that, after varising DFO for unicompartmental OA, 14 out of 15 patients (93%) returned to their preoperative activity level and 23 out of 26 patients (89%) returned to work [4]. The authors found a median Tegner score of 3.0 (range 1–7) both pre- and postoperatively, compared to a median Tegner score of 4.0 (range 0–10) pre-symptomatically and 3.0 (range 0–10) postoperatively in the present cohort. Thus, RTS was slightly higher in De Carvalho’s cohort, while the Tegner score was higher in the present study. This difference cannot be explained by age distribution, which was similar in both groups, or surgical indication, since subgroup analysis of the OA group in the present study showed similar findings. Thus, no clear reason could be identified for the difference between both studies. Another study investigated RTS in 13 young athletes participating in high-impact sports ≥ 4 times per week. All athletes returned to their prior level, which is a promising finding, indicating that even a return to high levels of athletic activity is possible after DFO [31].

Finally, finding the optimal treatment strategy for the increasing number of young patients with “old knees”, who tend to have expectations that exceed the improvements a knee arthroplasty can deliver [1, 24], remains challenging. According to the algorithm proposed by Arnold et al., the highest priority in any affected knee should be a balanced mechanical leg axis [1]. Due to the high variety of indications and broad age range in our study population, our results are likely more generalizable to the total DFO population than previously reported results in young athletes and lateral OA patients [4, 31]. Consequently, these findings can be of use for shared decision making in a broader DFO population. The general view arising from current limited literature is that RTS and RTW after DFO is possible and might even be higher compared to surgical alternatives such as TKA and UKA.

An important limitation of the present study is the retrospective design, which makes our findings prone to recall bias. Future prospective studies are needed to control for this aspect and to further elaborate on the fulfilment of patients’ expectations after DFO. In addition, no validated questionnaire exists to ascertain participation in sport and work. To improve comparability, a sports questionnaire was used that has been previously described in patients undergoing TKA, UKA and HTO [8, 22, 23], and the validated Tegner and Lysholm score were added. For the work-related outcomes, the validated WORQ questionnaire was used to increase reliability and validity of our findings [9, 18].

## Conclusion

In conclusion, almost eight out of ten patients return to sport and nine out of ten patients return to work after DFO. These are clinically relevant findings that further justify DFO as a surgical alternative to KA in young, active knee OA patients who wish to return to high activity levels.

## Electronic supplementary material

Below is the link to the electronic supplementary material.

Supplementary material 1 (DOCX 15 KB)

Supplementary material 2 (DOCX 15 KB)
