# Supplementary material for: Eight respectively nine out of ten patients return to sport and work after distal femoral osteotomy
Source: Knee Surg Sports Traumatol Arthrosc. 2018 Oct 22;27(7):2345–53. doi: 10.1007/s00167-018-5206-x (PMC6609586; doi:10.1007/s00167-018-5206-x)
Supplement: Supplementary file 1 — Supplementary material 1 (DOCX 15 KB) [file 167_2018_5206_MOESM1_ESM.docx]

**Additional file 1** Low-, intermediate- and high-impact sports activities included in the questionnaire and total number of participants for each sport at four time points

| **Level of impact*** | **Sport** | **Pre-symptomatic** | **1 year preoperatively** | **1 year postoperatively** | **At final follow-up** |
| --- | --- | --- | --- | --- | --- |
| Low | Nordic walking | 2 | 1 | 2 | 2 |
| Low | Cycling | 63 | 54 | 52 | 60 |
| Low | Road bicycle racing | 9 | 5 | 6 | 9 |
| Low | Swimming | 42 | 26 | 30 | 29 |
| Low | Aqua aerobics | 6 | 3 | 2 | 3 |
| Low | Cross-country skiing | 2 | 1 | 1 | 1 |
| Low | Golf | 4 | 2 | 2 | 4 |
| Low | Table tennis | 4 | 1 | 1 | 0 |
| Low | Dancing | 15 | 5 | 3 | 7 |
| Low | Sailing | 2 | 0 | 0 | 1 |
| Low | Rowing | 0 | 0 | 0 | 0 |
| Intermediate | Inline skating | 9 | 5 | 4 | 5 |
| Intermediate | Hiking | 16 | 4 | 5 | 7 |
| Intermediate | Mountain climbing | 2 | 1 | 0 | 0 |
| Intermediate | Mountain biking | 8 | 5 | 4 | 5 |
| Intermediate | Fitness-/weight-training | 39 | 33 | 35 | 39 |
| Intermediate | Aerobics | 10 | 5 | 2 | 5 |
| Intermediate | Gymnastics | 7 | 1 | 2 | 3 |
| Intermediate | Downhill skiing | 16 | 4 | 6 | 6 |
| Intermediate | Snowboarding | 4 | 2 | 2 | 3 |
| Intermediate | Ice skating | 23 | 5 | 3 | 5 |
| Intermediate | Tennis (doubles) | 14 | 6 | 5 | 6 |
| Intermediate | Horse riding | 7 | 0 | 1 | 0 |
| High | Jogging | 33 | 9 | 7 | 7 |
| High | Ice hockey | 0 | 0 | 0 | 0 |
| High | Tennis (singles) | 17 | 3 | 1 | 0 |
| High | Squash | 2 | 0 | 0 | 0 |
| High | Badminton | 5 | 0 | 1 | 1 |
| High | Soccer | 22 | 7 | 4 | 1 |
| High | Handball | 4 | 1 | 0 | 0 |
| High | Volleyball | 7 | 2 | 1 | 1 |
| High | Baseball | 0 | 0 | 0 | 0 |
| High | Martial arts | 11 | 4 | 2 | 1 |
| High | Water skiing | 1 | 1 | 1 | 1 |
| High | Basketball | 4 | 0 | 0 | 0 |

*Level of impact according to Vail et al. [28]
