# Supplementary material for: Eight respectively nine out of ten patients return to sport and work after distal femoral osteotomy
Source: Knee Surg Sports Traumatol Arthrosc. 2018 Oct 22;27(7):2345–53. doi: 10.1007/s00167-018-5206-x (PMC6609586; doi:10.1007/s00167-018-5206-x)
Supplement: Supplementary file 2 — Supplementary material 2 (DOCX 15 KB) [file 167_2018_5206_MOESM2_ESM.docx]

**Additional file 2** Sports frequency, level of impact and time to RTS of the total group at four time points*

|  | **Pre-symptomatically** (n (%)) | **1 year pre-operatively**  (n (%)) | **1 year post-operatively**  (n (%)) | **At final follow-up**  (n (%)) |
| --- | --- | --- | --- | --- |
| **Sports frequency,** times/wk* |  |  |  |  |
| No participation | 16 (16) | 36 (37) | 41 (42) | 31 (31) |
| ≤1 | 17 (21) | 24 (25) | 19 (19) | 23 (24) |
| 2 | 24 (29) | 23 (23) | 24 (25) | 18 (19) |
| 3 | 21 (26) | 10 (10) | 11 (11) | 17 (17) |
| ≥4 | 20 (24) | 5 (5) | 3 (3) | 9 (9) |
| **Sports participation,** hrs/wk |  |  |  |  |
| No participation | 16 (16) | 37 (37) | 41 (42) | 31 (31) |
| 0 – 2 | 25 (25) | 30 (31) | 28 (28) | 28 (28) |
| 3 – 4 | 18 (18) | 19 (19) | 19 (19) | 27 (27) |
| 5 – 6 | 23 (23) | 9 (9) | 9 (9) | 8 (8) |
| >6 | 17 (17) | 4 (4) | 2 (2) | 6 (6) |
| **Level of impact** |  |  |  |  |
| Low | 149 (36) | 98 (50) | 99 (54) | 116 (55) |
| Intermediate | 155 (38) | 71 (36) | 69 (37) | 84 (39) |
| High | 106 (26) | 27 (14) | 17 (9) | 12 (6) |
| **Total sports** | **410 (–)** | **196 (–)** | **185 (–)** | **212 (–)** |
| **Time to RTS,** wks |  |  |  |  |
| 0 – 12 | – | – | – | 16 (19) |
| 13 – 15 | – | – | – | 19 (23) |
| 16 – 18 | – | – | – | 7 (8) |
| 19 – 22 | – | – | – | 4 (5) |
| 23 – 26 | – | – | – | 4 (5) |
| >26 | – | – | – | 20 (24) |
| Did not RTS | – | – | – | 13 (16) |

hrs: hours, RTS: return to sport, wk: week
* Number of patients differs from the sports participation (hrs/wk) due to one case with missing values for sports frequency at one year pre- and postoperatively and final follow-up.
